# Clinical and Genomic Characterization of High-Risk Multidrug-Resistant *Klebsiella pneumoniae* Lineages in Pakistan

**DOI:** 10.3390/microorganisms14071462

**Published:** 2026-07-02

**Authors:** Aakash Ahmad Khattak, Sadiq Azam, Noor Rehman, Muhammad Asghar, Aiman Waheed, Sajjad Ahmad, Jody E. Phelan, Susana Campino, Taj Ali Khan, Taane G. Clark

**Affiliations:** 1 Centre of Biotechnology and Microbiology, University of Peshawar, Peshawar 25120, Pakistan; akash.khattak1@gmail.com (A.A.K.);; 2 Department of Pathology, Khyber Teaching Hospital, Peshawar 25120, Pakistan; noorbangash61@gmail.com (N.R.); drazghar@gmail.com (M.A.); 3 Institute of Pathology and Diagnostic Medicine, Khyber Medical University, Peshawar 25100, Pakistan; draimanwaheed.ibms@kmu.edu.pk (A.W.); sajjadahmad.ibms@kmu.edu.pk (S.A.); 4 Faculty of Infectious and Tropical Diseases, London School of Hygiene and Tropical Medicine, London WC1E 7HT, UK; jody.phelan@lshtm.ac.uk (J.E.P.); susana.campino@lshtm.ac.uk (S.C.); 5 Faculty of Epidemiology and Population Health, London School of Hygiene and Tropical Medicine, London WC1E7HT, UK

**Keywords:** *K. pneumoniae*, multidrug resistance, whole-genome sequencing, antibiotic resistance genes, chromosomal mutations, phylogenetic analysis

## Abstract

Multidrug-resistant (MDR) *Klebsiella pneumoniae* represents a major clinical and public health challenge worldwide, particularly in regions with limited genomic surveillance. This study investigated the clinical, phenotypic, and genomic characteristics of clinical *K. pneumoniae* isolates recovered from a tertiary-care hospital in Peshawar, Pakistan. A total of 2400 non-duplicate clinical specimens were processed, and antimicrobial susceptibility testing was performed according to CLSI guidelines. Whole-genome sequencing (WGS) was conducted on a purposively selected subset of 18 isolates representing diverse resistance and phenotypic profiles. Genomic analyses included multilocus sequence typing, resistome and virulome profiling, identification of resistance-associated chromosomal mutations, plasmid replicon typing, and phylogenomic comparison with publicly available international genomes. *K. pneumoniae* was identified in 256/2400 (10.7%) specimens, predominantly from urine samples. MDR and extensively drug-resistant (XDR) phenotypes were detected in 83.2% and 13.3% of isolates, respectively. WGS revealed substantial genomic heterogeneity, with ST147 identified as the most frequent lineage among sequenced isolates. Extended-spectrum β-lactamase genes, particularly *bla*_CTX-M-15_, together with carbapenemase genes including *bla*_OXA-48_-like and *bla*_NDM-5_, were identified in multiple isolates alongside resistance-associated chromosomal alterations in *gyrA*, *parC*, *ompK36*, *mgrB*, *pmrB*, and *ramR*. Yersiniabactin-associated loci were detected in all sequenced isolates, whereas canonical hypervirulence-associated determinants *(rmpA, iuc, iro*) were absent. These findings highlight the complex genomic landscape of MDR *K. pneumoniae* in Pakistan and underscore the need for continued genomic surveillance and antimicrobial stewardship.

## 1. Introduction

*Klebsiella pneumoniae* (*K. pneumoniae)* is a significant Gram-negative opportunistic pathogen that causes a broad range of healthcare- and community-associated infections, such as urinary tract infections, pneumonia, bloodstream infections, and surgical site infections [1]. Its clinical significance has increased significantly in the last 2 decades because of the rapid development and spread of antimicrobial resistance, especially extended-spectrum beta-lactamases (ESBLs) and carbapenemases [2]. The World Health Organization has designated carbapenem-resistant Enterobacterales, including carbapenem-resistant *K. pneumoniae*, as critical-priority pathogens because of their association with limited therapeutic options, increased healthcare burden, and elevated mortality rates [3].

The global expansion of MDR *K. pneumoniae* is driven by a combination of clonal dissemination, acquisition of mobile antimicrobial resistance determinants, and adaptive chromosomal evolution under sustained antimicrobial selection pressure [4]. High-risk sequence types (STs), including ST147, ST307, ST11, and ST258, have been repeatedly associated with nosocomial outbreaks, carbapenem resistance, and international dissemination. These lineages frequently harbor plasmid-associated resistance determinants carried by incompatibility groups such as IncF and IncL/M, which facilitate the spread of antimicrobial resistance genes across genetically diverse bacterial populations [5].

In addition to antimicrobial resistance determinants, *K. pneumoniae* possesses multiple virulence-associated factors involved in colonization, persistence, immune evasion, and host adaptation. Among these, siderophore-mediated iron acquisition systems, including enterobactin, yersiniabactin, aerobactin, and salmochelin, are important for bacterial survival under host-imposed iron limitation. Yersiniabactin-associated loci are increasingly detected in both classical and MDR *K. pneumoniae* lineages and may contribute to enhanced bacterial fitness during colonization and infection. However, the presence of individual siderophore loci alone does not establish a hypervirulent phenotype. Additional virulence-associated features, including capsular polysaccharide biosynthesis loci, fimbrial adhesins, and secretion-associated systems, further contribute to pathogenic potential and ecological adaptation [6].

Historically, hypervirulent and MDR *K. pneumoniae* populations were viewed as separate, with hypervirulent strains having canonical markers, including *rmpA, iuc* (aerobactin), and *iro* (salmochelin), which are commonly linked to hypermucoviscosity and invasive disease phenotypes [7]. However, recent genomic studies indicate increasing overlap between resistance-associated and virulence-associated genetic features in certain lineages. Importantly, hypermucoviscosity alone is not considered a definitive surrogate marker of hypervirulence, and the absence or presence of individual virulence loci should be interpreted cautiously within broader genomic and phenotypic contexts [8].

South Asia is a significant epicenter of antimicrobial resistance because of the high use of antibiotics, weak regulatory controls, and highly interconnected healthcare systems that promote the spread of pathogens [9,10]. The incidence of ESBL- and carbapenemase-producing *K. pneumoniae* has been reported to rise in Pakistan, with some strains harboring *bla*_OXA-48_ like and *bla*_NDM_ variants [11]. Despite these reports, comprehensive genomic investigations integrating resistome profiling, chromosomal resistance-associated mutations, virulence-associated loci, plasmid replicon diversity, and phylogenomic context remain limited in Pakistan and neighboring regions [12].

Whole-genome sequencing (WGS) has emerged as a powerful technique of high-resolution characterization of bacterial pathogens, enabling the concomitant interrogation of population structure, antimicrobial resistance determinants, mobile genetic elements, and evolutionary dynamics [13]. In addition to the identification of acquired resistance genes, WGS enables the identification of chromosomal mutations in important loci, such as *ompK36*, *mgrB*, *pmrB*, *ramR*, *gyrA*, and *parC*, which are associated with decreased susceptibility to carbapenems, colistin, and fluoroquinolones. Nevertheless, interpretation of genomic findings requires caution, particularly when based on purposively selected isolate subsets and short-read sequencing approaches that do not fully resolve plasmid architecture or mobile genetic element organization [14].

In the present study, we performed an integrated clinical, phenotypic, and genomic characterization of clinical *K. pneumoniae* isolates recovered from a tertiary-care hospital in Peshawar, Pakistan. Specifically, the objectives of this study were to: (i) determine the prevalence and antimicrobial resistance profiles of clinical *K. pneumoniae* isolates; (ii) characterize the genomic diversity and sequence type distribution among selected MDR/XDR isolates; (iii) identify acquired antimicrobial resistance genes and resistance-associated chromosomal alterations; (iv) investigate the distribution of virulence-associated loci, particularly siderophore-associated determinants; and (v) examine plasmid replicon diversity and phylogenomic relatedness in comparison with publicly available international genomes. By integrating phenotypic and genomic data, this study provides additional insight into the molecular epidemiology of MDR *K. pneumoniae* circulating in a high-burden healthcare setting while acknowledging the methodological limitations inherent to the sequencing strategy employed.

## 2. Materials and Methods

### 2.1. Study Design, Ethical Approval, and Study Setting

This cross-sectional observational study was conducted at Khyber Teaching Hospital (KTH), Peshawar, Pakistan, a major tertiary-care referral hospital serving a large population within Khyber Pakhtunkhwa province. Clinical specimens were collected between February 2023 and December 2024 as part of routine diagnostic microbiology services. The study protocol was approved by the Institutional Research and Ethical Review Board of Khyber Medical College and Khyber Teaching Hospital (Approval No. 115/DME/KMC; approved on 22 February 2024). All patient identifiers were anonymized prior to analysis, and no direct patient intervention was performed.

### 2.2. Clinical Specimens and Bacterial Isolation

A total of 2400 non-duplicate clinical specimens, such as urine, blood, respiratory samples, wound swabs, and other body fluids, were collected. Specimens were collected according to clinical indications rather than sequential enrollment, reflecting routine hospital diagnostic practices. Patients receiving active antimicrobial therapy at the time of sample collection were excluded when clinically feasible to reduce culture suppression bias [12].

Blood samples were inoculated in REDOX™ aerobic culture bottles and incubated using VersaTrek ™ automated system (Thermo Fisher Scientific, Waltham, MA, USA) up to 7 days [15]. Other specimens were cultured on MacConkey agar, blood agar, and cystine-lactose-electrolyte-deficient (CLED) agar and incubated aerobically at 37 C 18–24 h [16]. Identification of the isolates of *K. pneumoniae* was performed using colony morphology (large, mucoid, lactose-fermenting pink colonies on MacConkey agar), Gram-negative bacillary morphology, and biochemical features [17]. Definitive identification was done using the API 20E system (bioMerieux, Marcy-l’Étoile, France). Only one non-duplicate isolate per patient was included in the analysis [12].

### 2.3. Antimicrobial Susceptibility Testing

The Kirby Bauer disk diffusion method was used to perform antimicrobial susceptibility testing (AST) on Mueller-Hinton agar and was interpreted according to the Clinical and Laboratory Standards Institute (CLSI) M100, 32nd Edition (2022), which represented the contemporaneous guideline available during the study period [18]. Bacterial suspensions were adjusted to a 0.5 McFarland turbidity (about 1.5 × 10^8^ CFU/mL). The antimicrobial panel consisted of β-lactams (amoxicillin-clavulanate, piperacillin-tazobactam), cephalosporins (ceftriaxime, ceftaxone, ceftazidime, cefepime), carbapenems (meropenem), aminoglycosides (gentamicin, amikacin), fluoroquinolones (ciprofloxacin), tetracyclines (tigecycline), nitrofurans (nitrofurantoin), and folate pathway inhibitors (trimethoprim–sulfamethoxazole).

Broth microdilution (ComASP™, Liofilchem, Roseto degli Abruzzi, Italy) was used to determine colistin and tigecycline minimum inhibitory concentrations (MICs) and interpreted based on guidelines that were in place at the time of the study [12]. The MDR and XDR phenotypes were defined using the European Centre for Disease Prevention and Control (ECDC) criteria based on non-susceptibility across antimicrobial classes rather than the absolute number of agents tested. MDR was defined as non-susceptibility to at least one agent in three or more antimicrobial categories, while XDR was defined as non-susceptibility to all but two or fewer antimicrobial categories tested [19].

### 2.4. Hypermucoviscosity Assessment

The string test was used to measure hypermucoviscosity. Colonies grown on blood agar were stretched using a sterile loop; formation of a viscous string ≥ 5 mm was considered positive. This test was not a definitive test of hypervirulence but a descriptive phenotypic characterization [20]. The string test was used solely as a descriptive phenotypic assessment and was not considered a definitive marker of hypervirulence.

### 2.5. Whole-Genome Sequencing

A purposive subset of 18 *K. pneumoniae* isolates was selected for whole-genome sequencing (WGS) to capture phenotypic and antimicrobial resistance diversity across the isolate collection. Selection criteria included MDR/XDR phenotypes, carbapenem resistance profiles, hypermucoviscosity phenotype, specimen source, and genomic DNA quality. Overnight cultures were used to extract genomic DNA using the GeneJET Genomic DNA Purification Kit (Thermo Fisher Scientific) according to the manufacturer’s instructions. DNA quality was evaluated by Qubit fluorometry, spectrophotometric purity (A260/A280 = 1.8–2.0), and agarose gel electrophoresis; all samples (*n* = 18) with adequate concentration (>20 ng/µL) and intact high-molecular-weight DNA were included for sequencing.

Sequencing libraries were prepared using the QIAseq FX DNA Library Kit (QIAGEN, Hilden, Germany) according to the manufacturer’s instructions. Paired-end sequencing (2 × 151 bp) was performed on the Illumina MiSeq platform with a target of about 100-fold genome coverage at the Applied Genomics Centre, London School of Hygiene and Tropical Medicine.

### 2.6. Bioinformatics Analysis and Genome Assembly

Raw sequencing reads were assessed for quality using FastQC (v0.12.1) [21], followed by adapter removal and quality trimming using fastp (v1.1.0) [22]. Quality metrics before and after trimming were summarized using MultiQC (v1.19) [23]. Taxonomic classification was performed using Kraken2 (v1.3.1), with visualization using Krona (v2.7.1) to confirm species identity [24]. The Sequence types of isolates were determined by multilocus sequence typing (MLST) targeting seven housekeeping genes [25].

High-quality reads were assembled de novo using SPAdes implemented within the Shovill pipeline (v1.4.2) [26]. Assembly quality metrics, including genome size, GC content, contig number, N50, and L50 values, were evaluated using QUAST software (v5.3.0). Genome annotation was performed via Prokka (v1.14.6) using *K. pneumoniae* reference strain HS11286 [accession no. CP003200] as a reference genome [27] and further validated using the RAST annotation server (https://bio.tools/rast, accessed on 22 June 2026) [28]. Assemblies were considered acceptable when genome size ranged between 5.0 and 7.0 Mb, GC content was approximately 56–58%, N50 exceeded 100 kb, contamination was not detected, and taxonomic assignment confirmed *K. pneumoniae*. All assemblies were retained after quality assessment and interpreted with consideration of assembly-specific variability [25].

### 2.7. Genomic Characterization of Resistance, Virulence, and Plasmids

Acquired antimicrobial resistance genes and resistance-associated chromosomal mutations were identified using ResFinder (v4.1) [29], the Comprehensive Antibiotic Resistance Database (CARD) [30], Kleborate (v2.3.2) [31], AMRFinderPlus (v3.12.8) [32], and ABRicate (v1.0.1) [29].

Virulence-associated genes were identified using the Virulence Factor Database (VFDB) [33] and Kleborate (v3.2.4) [31], with particular emphasis on siderophore-associated loci and canonical hypervirulence-associated determinants including *rmpA/rmpA2, iuc,* and *iro*. Plasmid replicon types were identified using PlasmidFinder (v2.1) [34].

### 2.8. Phylogenomic Analysis

Phylogenomic reconstruction was performed using the Bacterial Genome Tree Service implemented in the Bacterial and Viral Bioinformatics Resource Center (BV-BRC). The codon-tree approach was used for phylogenetic inference, whereby single-copy protein families (PGFams) shared among selected genomes were identified and aligned using conserved coding sequences. Maximum-likelihood phylogenetic trees were generated using RAxML (v2.0.2) based on concatenated alignments of single-copy orthologous genes [35,36].

The reference genome *K. pneumoniae* HS11286 was included for comparative purposes. Branch support was assessed using 1000 bootstrap replicates, and phylogenetic trees were visualized and annotated using Interactive Tree of Life (iTOL) (v7) (https://itol.embl.de/).

For global phylogenomic contextualization, publicly available *K. pneumoniae* genomes representing diverse geographical regions and sequence types were selected from the BV-BRC public genome repository. Genome selection prioritized high-quality assemblies and sequence types corresponding to those identified in the present study, particularly ST147-associated isolates. The resulting phylogenetic analysis was intended to assess genomic relatedness rather than infer direct transmission events or epidemiological linkage.

### 2.9. Statistical Analysis

IBM SPSS (v23) was used to conduct statistical analyses. Chi-square or Fisher exact test was used to compare categorical variables. Logistic regression was used to identify the factors that were associated with *K. pneumoniae* infection. Adjusted (multivariate) odds ratios (aORs) and unadjusted (univariate) odds ratios (ORs), and 95% confidence intervals (CIs) were calculated. A *p*-value < 0.05 was considered statistically significant [37].

## 3. Results

### 3.1. Prevalence and Clinical Distribution of K. pneumoniae

Among the 2400 non-duplicate clinical specimens processed during the study period, *K. pneumoniae* was isolated from 256 samples, corresponding to an overall prevalence of 10.7%. Urine specimens constituted the predominant source of isolation, accounting for 152/256 (59.4%) of all isolates, followed by surgical site infection specimens (64/256, 25.0%), respiratory specimens (24/256, 9.4%), and blood culture isolates (16/256, 6.3%) (Figure S1).

Demographic analysis demonstrated that 164/256 (64.1%) isolates were collected from female patients, whereas 92/256 (35.9%) were collected from male patients (Table S1). The higher proportion of female cases was primarily attributable to the predominance of urinary tract isolates, which represented the most frequent clinical source of *K. pneumoniae*.

Geographically, most of the isolates originated from patients residing in Peshawar (172/256, 67.2%), followed by Abbottabad (40/256, 15.6%), whereas the remaining districts collectively accounted for a smaller proportion of cases (Table S1). Age-stratified analysis indicated that the highest number of *K. pneumoniae* infections occurred among patients aged 21–30 years (64/256, 25.0%), followed by those aged 41–50 years (32/256, 12.5%), 51–60 years (36/256, 14.1%), and 61–70 years (36/256, 14.1%). No distinct bimodal age distribution was observed (Table S1).

### 3.2. Antimicrobial Resistance Profiles

AST revealed that the burden of resistance to different classes of antibiotics was high. The prevalence of resistance to β-lactam agents was high, with over 70 percent of isolates resistant to amoxicillin-clavulanate and 45–59 percent resistant to third-generation cephalosporins (ceftaxime, ceftriaxone, ceftazidime). The resistance to cefepime was also high, which means that it affected the efficacy of fourth-generation cephalosporins. Carbapenem resistance, as measured by meropenem, was found in 25 percent of isolates, which is a serious loss of last-line treatment. Resistance to fluoroquinolones was widespread, which is in line with the high selection pressure in clinical practice. Amikacin was relatively more active than gentamicin among aminoglycosides, but the resistance was still high. On the other hand, tigecycline and nitrofurantoin were the most active in vitro, and their susceptibility rates exceeded 85%, indicating that they are still therapeutically useful in certain clinical conditions (Table 1). Using standardized ECDC/CDC definitions, 213 (83.2%) isolates were found to be MDR, and 34 (13.3%) were classified as XDR.

### 3.3. Clinical and Laboratory Correlates

Univariate analysis identified significant associations between *K. pneumoniae* infection and female sex, selected age groups, residence in Peshawar, leukocytosis, neutrophilia, elevated C-reactive protein (CRP), elevated serum urea, elevated serum creatinine, diabetes mellitus, chronic kidney disease, coronary heart disease, and interstitial lung disease (Table S2). Variables demonstrating statistical significance in univariate analyses were subsequently included in the multivariable logistic regression model.

Following adjustment for potential confounders, female sex (aOR 1.65, 95% CI 1.25–2.19; *p* < 0.001), age groups 21–30 years (aOR 1.41, 95% CI 1.02–1.95; *p* = 0.036), 51–60 years (aOR 1.48, 95% CI 1.08–2.03; *p* = 0.014), and 61–70 years (aOR 1.45, 95% CI 1.04–2.02; *p* = 0.027), residence in Peshawar (aOR 1.43, 95% CI 1.10–1.86; *p* = 0.006), leukocytosis (aOR 2.25, 95% CI 1.55–3.25; *p* < 0.001), neutrophilia (aOR 3.95, 95% CI 2.71–5.76; *p* < 0.001), elevated CRP (aOR 14.50, 95% CI 7.20–29.20; *p* < 0.001), elevated serum urea (aOR 1.55, 95% CI 1.12–2.17; *p* = 0.008), elevated serum creatinine (aOR 1.48, 95% CI 1.06–2.07; *p* = 0.019), and diabetes mellitus (aOR 1.52, 95% CI 1.10–2.10; *p* = 0.012) remained independently associated with *K. pneumoniae* infection. Chronic kidney disease (aOR 0.68, 95% CI 0.48–0.97; *p* = 0.032) and interstitial lung disease (aOR 0.38, 95% CI 0.24–0.61; *p* < 0.001) also retained statistical significance following multivariable adjustment, whereas hypertension, coronary heart disease, elevated alanine aminotransferase (ALT), and residence in Abbottabad were not independently associated with infection.

### 3.4. Whole-Genome Sequencing and Assembly Quality

WGS was performed on 18 *K. pneumoniae* isolates representing diverse antimicrobial resistance and phenotypic profiles (Table S3). The sequencing subset included 10 MDR and 8 XDR isolates selected to maximize genomic diversity rather than to estimate population-level prevalence. The virulence and resistance scores calculated by Kleborate are displayed in Table S3. Following quality control, all assemblies met predefined quality criteria and were retained for downstream analyses. Genome sizes ranged from approximately 5.1 to 5.6 Mb, with GC contents consistent with *K. pneumoniae*. Assembly metrics, including contig counts, N50 values, and predicted coding sequence numbers, are summarized in Table S4.

### 3.5. Population Structure and Phylogenomic Relationships

MLST revealed a great degree of genetic heterogeneity, and multiple sequence types were found across the dataset. Seven STs were identified among the 18 genomes, including ST147 (*n* = 6), ST37 (*n* = 3), ST2629 (*n* = 3), ST151 (*n* = 1), ST870 (*n* = 1), ST1310 (*n* = 1), and ST45 (*n* = 1) (Table S3). ST147 was the most frequently identified sequence type within the sequenced subset. Because WGS was performed on a purposively selected subset of isolates, these ST frequencies should not be interpreted as representative of the overall population structure of all 256 clinical isolates.

**Figure 1:**
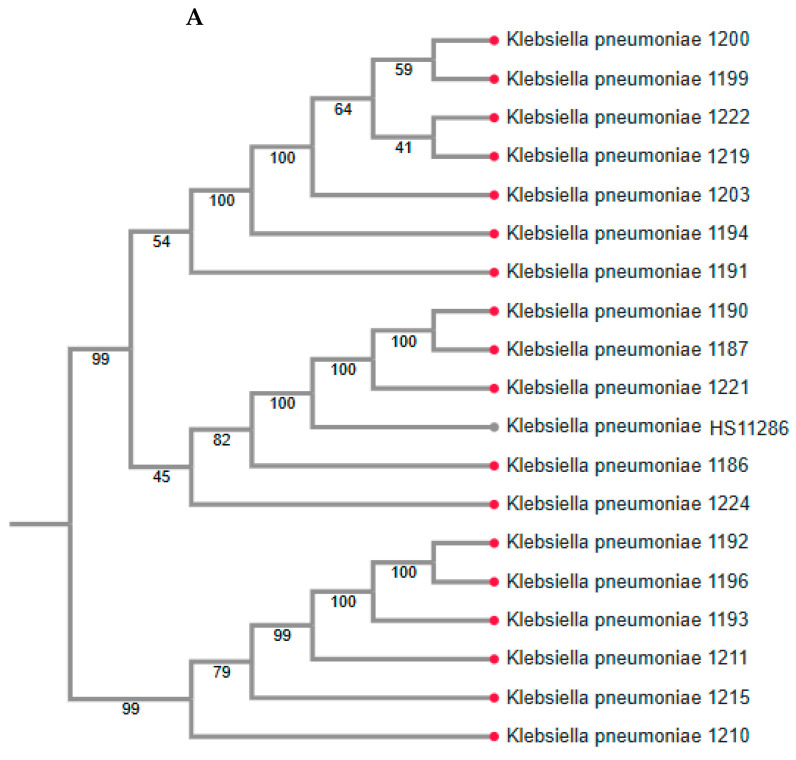

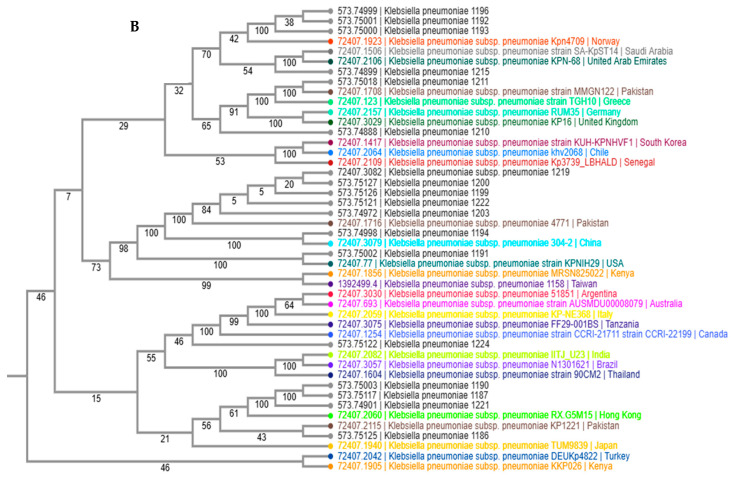
(**A**): Phylogenetic tree showing genetic relationships among 19 *K. pneumoniae* isolates. HS11286 (gray node) was used as the reference strain; the remaining 18 were clinical isolates (Red node). Closely related pairs include 1199–1200, 1222–1219, 1187–1221, 1192–1196, 1193–1211, and 1215–1210. Diverse isolates such as 1203, 1194, 1191, 1190, 1186, and 1224 show greater genetic divergence. (**B**). The phylogenetic tree of 18 *K. pneumoniae* study isolates labeled in *bla*ck in association with earlier reported Global samples (2020–2023); having different colors for each isolate; Saudi Arabia, Norway, Kenya, USA, Canada, Chile, UAE, Greece, China, India, Taiwan, Japan, Turkey, Germany, UK, Pakistan and Brazil. Closely related local isolates include 1192–1196, 1193–1211, 1187–1221, and 1199–1200, forming high-bootstrap clusters with minimal divergence. Some local strains (e.g., 1224, 1186, 1210 and 1194) formed distinct clades, reflecting significant genetic diversity. Several Pakistani isolates clustered with strains from the UAE, China, and Greece, indicating genomic relatedness within broader international phylogenetic groups. These observations should not be interpreted as evidence of direct transmission events or epidemiological linkage.

Core genome phylogenetic analysis revealed considerable genomic diversity among the study isolates (Figure 1A). Isolates sharing the same sequence type generally clustered together, although phylogenetic support varied across the tree. Strongly supported clusters included Kp1187–Kp1190 and Kp1192–Kp1196 (bootstrap = 100), whereas relationships among several other isolates were supported by lower bootstrap values and should therefore be interpreted with caution.

For the global phylogenomic context, the study isolates were analyzed alongside publicly available international genomes representing diverse geographical regions and sequence types (Figure 1B). Several Pakistani isolates clustered within broader phylogenetic groups containing isolates from Asia, Europe, North America, Africa, and the Middle East. However, as multiple internal branches exhibited low bootstrap support, these findings indicate broad genomic relatedness rather than direct epidemiological linkage or transmission events.

### 3.6. Virulence-Associated Gene Repertoire

**Figure 2:**
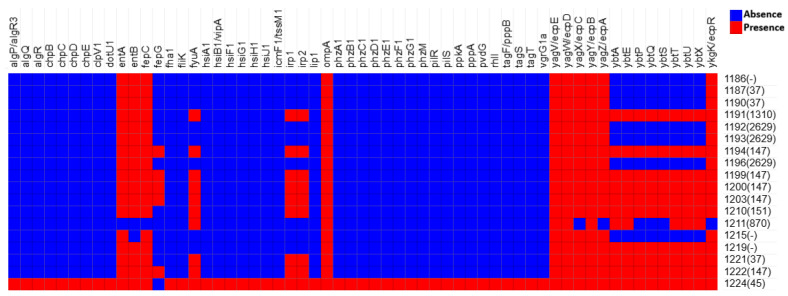
The Heatmap shows virulent genes among different isolates. Rows represent individual isolates, and columns represent virulence-associated genes identified through genomic analysis. Isolates are labeled with their corresponding STs in parentheses (a hyphen means that the ST could not be determined). Color intensity indicates the presence or absence of the respective virulence-associated genes, as shown in the legend. Hierarchical clustering was performed based on virulence gene profiles.

Virulence-associated gene analysis identified several conserved determinants among the sequenced *K. pneumoniae* isolates (Figure 2). Enterobactin-associated genes were detected in most genomes, whereas yersiniabactin-associated loci were identified in all sequenced isolates. At least one component of the yersiniabactin locus was detected in all isolates; however, the complete gene repertoire varied among genomes.

In contrast, canonical hypervirulence-associated determinants, including *rmpA*, i*uc* (aerobactin), and *iro* (salmochelin), were not detected in any of the sequenced isolates. These findings indicate that the analyzed isolates possessed siderophore-associated iron acquisition systems but lacked the molecular markers commonly associated with classical hypervirulent *K. pneumoniae* lineages.

Hypermucoviscosity, as determined by the string test, was observed in a subset of isolates, including Kp1200, Kp1199, Kp1186, Kp1187, Kp1190, Kp1191, Kp1192, Kp1193, Kp1196, and Kp1194. However, this phenotype was not accompanied by the presence of canonical hypervirulence-associated genes, suggesting discordance between hypermucoviscosity and established genomic markers of hypervirulence in the analyzed isolates.

### 3.7. Resistance-Associated Chromosomal Mutations and Resistome

**Figure 3:**
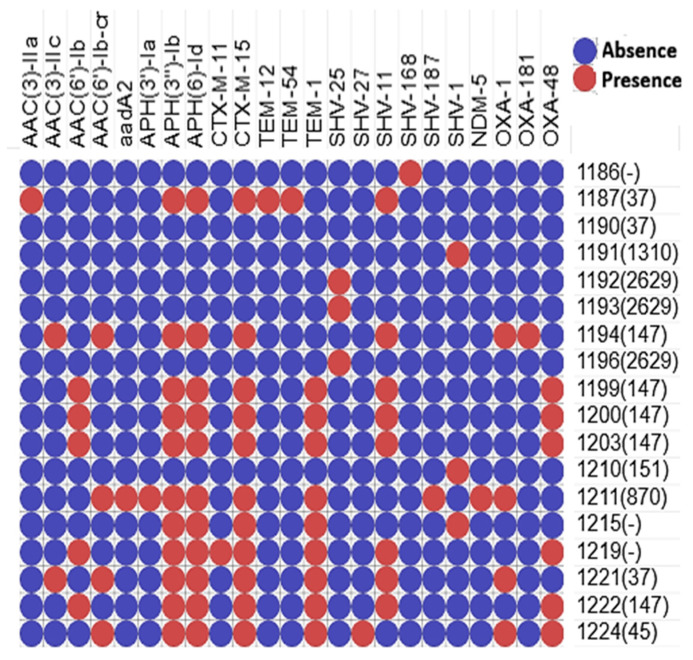
The heatmap illustrates the correlation between *K. pneumoniae* isolates IDs with their corresponding STs in brackets (a hyphen means that the ST could not be determined) (on the y-axis) and antimicrobial resistance (AMR) genes(on the x-axis). Blue color shows the presence of AMR genes in different clinical isolates, while a red color indicates the absence of AMR genes.

Comprehensive resistome analysis identified a diverse repertoire of acquired antimicrobial resistance determinants among the sequenced isolates (Figure 3). ESBL-associated gene *bla*_CTX-M-15_ was detected in 11 isolates, making it the most prevalent β-lactamase gene identified. Other β-lactamase genes included *bla*_SHV-1_ in 3, *bla*_SHV-11_ in 8, *bla*_SHV-27_ in 1, *bla*_TEM-54_ in 1, *bla*_TEM-1_ in 9, and *bla*_TEM-12_ in 1 isolate. Carbapenemase-associated genes were also detected, including *bla*_OXA-48_ in 6, *bla*_OXA-1_ in 3, *bla*_OXA-181_ in 1 and *bla*_NDM-5_ in 1 isolate. The presence of these carbapenemase genes was generally consistent with the observed phenotypic resistance to meropenem identified during antimicrobial susceptibility testing. Plasmid-mediated quinolone resistance genes, including *qnrB* and *qnrS*, were also detected in multiple isolates and may contribute to reduced fluoroquinolone susceptibility when present alongside chromosomal resistance-associated alterations.

In addition to acquired resistance genes, several chromosomal mutations previously associated with antimicrobial resistance were identified (Table S5). Alterations in *gyrA* and *parC*, including the well-described substitutions *gyrA* S83I and *parC* S80I, were detected in all sequenced isolates and have previously been associated with fluoroquinolone resistance and may contribute to the observed resistance phenotypes. Mutations in *omp*K36 were identified in multiple isolates, including N221H, Y201F, and H349R substitutions, which have previously been associated with reduced outer membrane permeability and decreased susceptibility to β-lactam agents, particularly when occurring in combination with ESBL or carbapenemase genes. Furthermore, mutations involving *mgrB, pmrB, arnC, lapB,* and *lpxM* were identified in several isolates. These genes are involved in lipopolysaccharide modification pathways and have previously been implicated in reduced colistin susceptibility. Similarly, a recurrent alteration in r*amR*, a regulator of multidrug efflux systems, was detected across the sequenced isolates and may contribute to multidrug-resistant phenotypes.

### 3.8. Plasmid Replicon Diversity and Resistance Dissemination

**Figure 4:**
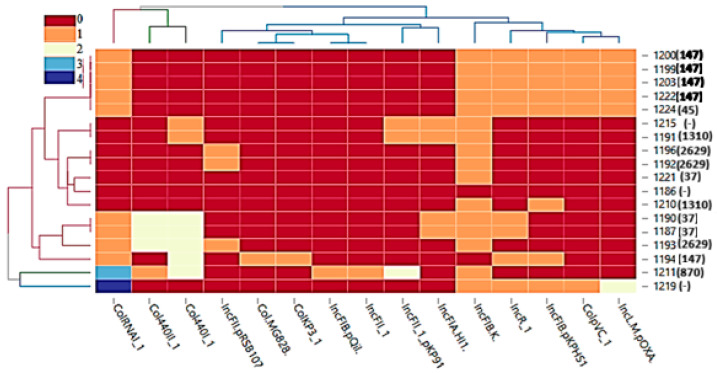
Heatmap of various plasmids among in-house isolates of *K. pneumoniae* (*n* = 18). Rows represent individual isolates, and columns represent plasmid replicons identified through genomic analysis. Isolates are labeled with their corresponding STs in parentheses (a hyphen means the ST could not be determined). Color intensity indicates the presence, absence or copy number of the respective plasmid replicon as shown in the legend.

Plasmid replicon analysis revealed considerable diversity among the sequenced *K. pneumoniae* isolates, with IncF and IncL/M replicons detected most frequently (Figure 4). Several isolates carried multiple plasmid replicon types simultaneously, indicating the presence of diverse plasmid backgrounds within individual genomes. IncF replicons frequently co-occurred with ESBL-associated resistance determinants, whereas IncL/M replicons were identified in several isolates carrying *OXA*-48-like carbapenemase genes. However, because plasmid analyses were based on short-read Illumina sequencing data, complete plasmid reconstruction and definitive assignment of antimicrobial resistance genes to specific plasmids were not possible. Consequently, the observed co-occurrence of plasmid replicons and resistance genes within the same isolates should not be interpreted as confirmed plasmid co-localization. Furthermore, no conjugation assays, plasmid transfer experiments, or long-read sequencing analyses were performed; therefore, conclusions regarding plasmid mobility, horizontal gene transfer, and resistance dissemination mechanisms remain inferential. Overall, these findings indicate the presence of diverse plasmid-associated genetic backgrounds among the sequenced isolates and underscore the need for plasmid-resolved genomic approaches to better characterize the genetic context of antimicrobial resistance determinants in local *K. pneumoniae* populations.

## 4. Discussion

The present study provides an integrated clinical, phenotypic, and genomic characterization of MDR *K. pneumoniae* clinical isolates recovered from a tertiary-care hospital in Pakistan. By combining antimicrobial susceptibility testing with WGS, the study investigated the distribution of resistance-associated determinants, sequence types, virulence-associated loci, chromosomal resistance-related alterations, and plasmid replicons among purposively selected MDR/XDR isolates. Importantly, the findings should be interpreted within the methodological constraints of a cross-sectional design, purposive isolate selection, and short-read sequencing–based genomic analysis.

The prevalence of MDR (83.2%) and XDR (13.3%) phenotypes in this cohort highlights the strength of antimicrobial selection pressure in the local healthcare setting. These rates are comparable or even greater than those in other South Asian and Middle Eastern settings, where ESBL- and carbapenemase-producing *K. pneumoniae* have become endemic [38,39,40]. The finding of carbapenem resistance in about a quarter of isolates is especially alarming, as carbapenems are used as the last-line therapy against severe Gram-negative infections, and alternative treatment options are scarce in resource-limited environments [41].

WGS demonstrated marked genomic heterogeneity among the sequenced isolates, with multiple STs identified across the dataset. ST147 represented the most frequently detected lineage within the purposively selected sequencing subset. ST147 is recognized internationally as a high-risk lineage frequently associated with ESBL production, carbapenem resistance, and healthcare-associated outbreaks [42,43,44]. However, because only 18 isolates underwent WGS and isolates were selected purposively to maximize phenotypic diversity, the observed sequence type distribution cannot be interpreted as representative of the broader clinical *K. pneumoniae* population within the hospital.

Phylogenomic analysis demonstrated clustering of several local isolates with publicly available international genomes, particularly within ST147-associated clades. Nevertheless, these findings should be interpreted cautiously. The phylogenetic trees generated in this study primarily reflect genomic relatedness and sequence similarity rather than confirmed transmission dynamics or direct epidemiological linkage. Furthermore, several branches demonstrated relatively low bootstrap support values, limiting the strength of inferences regarding fine-scale phylogenetic relationships. The inclusion of publicly available genomes was intended to provide a broader genomic context rather than establish international transmission pathways [12].

The resistome analysis identified widespread carriage of ESBL-associated genes, particularly *bla*_CTX-M-15_, together with additional β-lactamase genes including *bla*_SHV_ and *bla*_TEM_ variants. Carbapenemase-associated genes, including *bla*_OXA-48_-like and *bla*_NDM-5_, were detected in multiple isolates and were generally consistent with observed phenotypic carbapenem resistance profiles. Plasmid-mediated quinolone resistance genes, including *qnrB* and *qnrS*, were also identified. These findings are consistent with the increasing dissemination of multidrug-resistant Enterobacterales reported across South Asia and neighboring regions [44].

In addition to acquired resistance genes, this paper shows the ubiquitous role of chromosomal mutations in antimicrobial resistance phenotypes. Repeat mutations in *gyrA* and *parC* were found in all sequenced isolates, which is in line with well-established mechanisms of fluoroquinolone resistance [45]. Likewise, *ompK*36 mutations probably decrease outer membrane permeability, which enhances β-lactam and carbapenem resistance, especially when combined with β-lactamase expression [46]. Changes in *mgrB* and *pmrB*, which are major regulators of lipid A modification pathways, were also observed and are known to mediate decreased colistin susceptibility [47]. Mutations in *ramR*, a global regulator of efflux pump expression, further highlight the role of regulatory network perturbations in multidrug resistance [48]. However, interpretation of these chromosomal alterations requires caution because the present study did not include functional validation experiments, transcriptomic analyses, complementation studies, or antimicrobial resistance mechanistic assays. Consequently, the identified mutations should be interpreted as putative resistance-associated alterations inferred from genomic homology and previously published literature, rather than definitive causal mechanisms of resistance in the analyzed isolates.

Virulence-associated genomic analysis demonstrated widespread detection of siderophore-associated loci, particularly yersiniabactin-associated genes. Yersiniabactin contributes to iron acquisition under host-imposed iron limitation and has been associated with enhanced bacterial persistence and colonization capacity [49,50]. However, although yersiniabactin-associated loci were identified in all sequenced isolates, individual *ybt*-associated genes varied between genomes, and no functional siderophore activity assays were performed. Therefore, conclusions regarding enhanced bacterial fitness or virulence potential cannot be established directly from genomic data alone. Nevertheless, canonical hypervirulence markers, such as *rmpA, iuc,* and *iro*, were not present, which means that the isolates do not meet the molecular requirements of classical hypervirulent *K. pneumoniae* [7]. These findings are in line with the emerging evidence that virulence is a continuum with MDR lineages progressively acquiring selected fitness-related determinants without necessarily attaining complete hypervirulent phenotypes [51]. The discordance between hypermucoviscosity and virulence gene content further highlights the shortcomings of phenotypic assays in defining hypervirulence [52]. Hypermucoviscosity and hypervirulence are related but distinct phenotypes. Previous studies have shown that hypermucoviscosity may occur in isolates lacking *rmpA/rmpA2* and aerobactin loci, suggesting involvement of alternative capsular regulatory pathways and environmental modulation of capsule production [53].

Plasmid replicons analysis showed a high plasmid burden, and IncF and IncL/M replicons were frequently detected. IncF plasmids are the most famous vectors of ESBL dissemination, and IncL/M plasmids are closely associated with *OXA-48*-like carbapenemases [54]. Several isolates carried multiple plasmid replicons simultaneously, indicating a relatively high plasmid burden within individual genomes. However, important methodological limitations must be acknowledged regarding plasmid-related interpretations. Because short-read sequencing does not reliably reconstruct complete plasmids, co-localization of plasmid replicons and resistance genes could not be confirmed. Therefore, our findings demonstrate plasmid replicon diversity rather than definitive plasmid-mediated transmission of specific resistance determinants. Therefore, conclusions regarding horizontal dissemination mechanisms remain inferential and should be interpreted conservatively [55].

The clinical correlations in this study, especially with female sex and diabetes mellitus, are in line with the known epidemiological trends of *K. pneumoniae* infection, especially in urinary tract infections and compromised host defenses [56,57]. Laboratory indicators like leukocytosis and neutrophilia were highly correlated with infection, but these results are probably indicative of the host inflammatory response and should be viewed as such [58]. However, laboratory markers such as leukocytosis, elevated inflammatory parameters, and neutrophilia should be interpreted as nonspecific indicators of infection-associated inflammation rather than organism-specific biomarkers.

Several methodological limitations should be considered when interpreting the findings of this study. First, the cross-sectional study design precludes assessment of temporal transmission dynamics, longitudinal clonal evolution, or outbreak-related dissemination patterns. Second, WGS was performed on a limited, purposively selected subset of isolates designed to capture phenotypic diversity rather than provide population-level epidemiological representation. Consequently, the genomic findings cannot be generalized to the overall *K. pneumoniae* population circulating within the institution or region. Third, the use of short-read sequencing limited the accurate reconstruction of plasmid architecture, mobile genetic elements, structural rearrangements, and resistance gene localization. Fourth, resistance-associated mutations and virulence-associated loci were identified using bioinformatic prediction tools without experimental validation. Fifth, no transcriptomic, proteomic, siderophore activity, virulence phenotyping, or conjugation assays were performed to confirm predicted genomic functions. Finally, phylogenetic analyses were based on publicly available comparative genomes selected for contextual analysis and therefore should not be interpreted as evidence of confirmed epidemiological linkage or international transmission events.

Despite these limitations, the study contributes additional genomic epidemiological data from a region where comprehensive WGS-based surveillance of MDR *K. pneumoniae* remains limited. The coexistence of multiple acquired resistance determinants, resistance-associated chromosomal alterations, and diverse plasmid replicons among clinically important MDR/XDR isolates underscores the genomic complexity of contemporary *K. pneumoniae* populations in high-burden healthcare environments. Future investigations incorporating larger representative isolate collections, long-read sequencing technologies, plasmid-resolved genomics, and functional validation experiments will be important to better define resistance dissemination dynamics, virulence-associated mechanisms, and evolutionary adaptation in MDR *K. pneumoniae*.

## 5. Conclusions

This study provides a combined clinical, phenotypic, and genomic characterization of MDR and XDR *K. pneumoniae* clinical isolates recovered from a tertiary-care hospital in Pakistan. Whole-genome sequencing of a purposively selected subset of isolates revealed substantial genomic diversity, including the presence of high-risk lineages such as ST147, together with widespread ESBL- and carbapenemase-associated resistance genes, notably *bla*_CTX-M-15_, *bla*_OXA-48_ like, and *bla*_NDM-5_. Multiple resistance-associated chromosomal alterations and diverse plasmid replicons were also identified, highlighting the complex genetic basis of antimicrobial resistance in these isolates. Yersiniabactin-associated loci were detected in all sequenced genomes, whereas canonical hypervirulence-associated determinants (*rmpA, iuc,* and *iro*) were absent. However, the findings should be interpreted cautiously because sequencing was performed on a limited, purposively selected subset, short-read sequencing restricted plasmid resolution, and functional validation experiments were not performed. Despite these limitations, the study contributes important genomic epidemiological data from a region with limited genomic surveillance and underscores the need for continued genomic monitoring, strengthened antimicrobial stewardship, and enhanced infection prevention strategies to mitigate the spread of MDR *K. pneumoniae*.

## Figures and Tables

**Table 1:** AST profile of *K. pneumoniae* isolates (*n* = 256).

Antimicrobial Agent	Antimicrobial Class	Susceptible *n* (%)	Resistant *n* (%)
Amoxicillin–Clavulanate (AMC)	β-lactam/β-lactamase inhibitor	64 (25.0)	192 (75.0)
Cefoperazone–Sulbactam (SCF)	β-lactam/β-lactamase inhibitor	172 (67.2)	84 (32.8)
Piperacillin–Tazobactam (TZP)	β-lactam/β-lactamase inhibitor	180 (70.3)	76 (29.7)
Ceftazidime–Avibactam (CZA)	β-lactam/β-lactamase inhibitor	168 (65.6)	88 (34.4)
Cefepime (FEP)	Fourth-generation cephalosporin	140 (54.7)	116 (45.3)
Cefotaxime (CTX)	Third-generation cephalosporin	104 (40.6)	152 (59.4)
Ceftriaxone (CRO)	Third-generation cephalosporin	104 (40.6)	152 (59.4)
Ceftazidime (CAZ)	Third-generation cephalosporin	136 (53.1)	120 (46.9)
Meropenem (MEM)	Carbapenem	192 (75.0)	64 (25.0)
Gentamicin (CN)	Aminoglycoside	132 (51.6)	124 (48.4)
Amikacin (AK)	Aminoglycoside	164 (64.1)	92 (35.9)
Ciprofloxacin (CIP)	Fluoroquinolone	132 (51.6)	124 (48.4)
Tigecycline (TGC)	Glycylcycline	224 (87.5)	32 (12.5)
Tetracycline (TE)	Tetracycline	72 (28.1)	184 (71.9)
Nitrofurantoin (F)	Nitrofuran	237 (92.5)	19 (7.5)
Trimethoprim–Sulfamethoxazole (SXT)	Folate pathway inhibitor	137 (53.5)	119 (46.5)

## Data Availability

The whole-genome sequencing data presented in this study are openly available in the NCBI Sequence Read Archive (SRA) under BioProject accession number PRJNA1377269. Sequencing data for all 18 isolates analyzed in this study are available under accession numbers SRR37139978–SRR37139995. At the time of manuscript submission and peer review, these datasets remain under repository embargo and are scheduled for public release on 31 December 2026. Upon release, all FASTQ files will be freely accessible through the NCBI SRA, enabling independent verification and reuse of the sequencing data.

## Supplementary Materials

The following supporting information can be downloaded at: https://www.mdpi.com/article/10.3390/microorganisms14071462/s1. Figure S1: Various infections and sample types retrieved from *K. pneumoniae* isolates, Table S1: Correlation of Demographic and Comorbid Factors with *K. pneumoniae* Infection, Table S2: Factors associated with *K. pneumoniae* infection in the current study, Table S3. Clinical, phenotypic, and genomic characteristics of 18 *K. pneumoniae* isolates selected for WGS, Table S4: De novo genome assembly of *K. pneumoniae* isolates, Table S5: Mutational analysis of AMR genes resistant isolates of *K. pneumoniae*.

## References

[1] Ndlovu T., Kgosietsile L., Motshwarakgole P., Ndlovu S.I. (2023). Evaluation of potential factors influencing the dissemination of multidrug-resistant *Klebsiella pneumoniae* and alternative treatment strategies. Trop. Med. Infect. Dis..

[2] Sid Ahmed M.A., Hamid J.M., Hassan A.M., Abu Jarir S., Bashir Ibrahim E., Abdel Hadi H. (2024). Phenotypic and genotypic characterization of pan-drug-resistant *Klebsiella pneumoniae* isolated in Qatar. Antibiotics.

[3] Alvisi G., Curtoni A., Fonnesu R., Piazza A., Signoretto C., Piccinini G., Sassera D., Gaibani P. (2025). Epidemiology and genetic traits of carbapenemase-producing enterobacterales: A global threat to human health. Antibiotics.

[4] Yakubu B. (2026). Antimicrobial Resistant Factors in *Klebsiella pneumoniae* Strains Isolated From Urinary Tract Infections, Wound Infections, Hospital Wastewater, and Cervical Cancers From Ghana, Togo, and Benin. Int. J. Genom..

[5] Mendes G., Ramalho J.F., Bruschy-Fonseca A., Lito L., Duarte A., Melo-Cristino J., Caneiras C. (2022). Whole-genome sequencing enables molecular characterization of non-clonal group 258 high-risk clones (ST13, ST17, ST147 and ST307) among carbapenem-resistant *Klebsiella pneumoniae* from a tertiary university hospital centre in Portugal. Microorganisms.

[6] Nguyen T.N.T., Howells G., Short F.L. (2025). How *Klebsiella pneumoniae* controls its virulence. PLoS Pathog..

[7] Russo T.A., Alvarado C.L., Davies C.J., Drayer Z.J., Carlino-MacDonald U., Hutson A., Luo T.L., Martin M.J., Corey B.W., Moser K.A. (2024). Differentiation of hypervirulent and classical *Klebsiella pneumoniae* with acquired drug resistance. MBio.

[8] Beck K.L., Agarwal A., Laufer Halpin A., McDonald L.C., McKay S.L., Kent A.G., Kaufman J.H., Mukherjee V., Elkins C.A., Seabolt E. (2025). De novo virulence feature discovery and risk assessment in *Klebsiella pneumoniae* based on microbial genome vectorization. Commun. Biol..

[9] Mohindra K.S., Haseen F., Adams M. (2025). The social determinants of superbugs: Antimicrobial resistance in South Asia. Handbook on the Social Determinants of Health.

[10] Gandra S., Alvarez-Uria G., Turner P., Joshi J., Limmathurotsakul D., van Doorn H.R. (2020). Antimicrobial resistance surveillance in low-and middle-income countries: Progress and challenges in eight South Asian and Southeast Asian countries. Clin. Microbiol. Rev..

[11] Habib S. (2023). Prevalence of antibiotic resistance in *Klebsiella* spp. and *Escherichia coli* isolates from human, animal, and environment sources in Pakistan. Ph.D. Thesis.

[12] Waheed A., Khan S.A., Ahmad S., Phelan J.E., Rani G.F., Campino S., Khan T.A., Clark T.G. (2026). Genomic Insights into Chromosomal Colistin Resistance and Virulence–Resistance Convergence in MDR/XDR *Klebsiella pneumoniae* from Tertiary Hospitals in Peshawar, Pakistan. Pathogens.

[13] Oniciuc E.A., Likotrafiti E., Alvarez-Molina A., Prieto M., Santos J.A., Alvarez-Ordóñez A. (2018). The present and future of whole genome sequencing (WGS) and whole metagenome sequencing (WMS) for surveillance of antimicrobial resistant microorganisms and antimicrobial resistance genes across the food chain. Genes.

[14] Gentile B., Grottola A., Orlando G., Fregni Serpini G., Venturelli C., Meschiari M., Anselmo A., Fillo S., Fortunato A., Lista F. (2020). A retrospective whole-genome sequencing analysis of carbapenem and colistin-resistant *Klebsiella pneumoniae* nosocomial strains isolated during an MDR surveillance program. Antibiotics.

[15] Wang H.-Y., Wu W.-H., Chiang M.-C., Hsu K.-H., Lin S.-Y., Wu I.-H., Chu S.-M., Lu J.-J., Hsu J.-F. (2025). Analysis and comparison of the difference between two major blood culture system in the laboratory and neonatal intensive care unit. Pediatr. Neonatol..

[16] Vadivelu N., Parmar R.D., Shingala H., Mehta K.D. (2025). Comparison of chromogenic and cysteine lactose electrolyte deficient agar for identification of uropathogens in Gujarat, India. Afr. J. Lab. Med..

[17] Al-Masoudi K.I.A., Al-Janabi H.S.O., Al-Mousawi H.T.M. (2025). Isolation and Characterization of *Klebsiella pneumoniae* from Urinary Tract Infections: A Comparative Study of Diagnostic Methods. SAR J. Pathol. Microbiol..

[18] Gaur P., Hada V., Rath R.S., Mohanty A., Singh P., Rukadikar A. (2023). Interpretation of antimicrobial susceptibility testing using European Committee on Antimicrobial Susceptibility Testing (EUCAST) and Clinical and Laboratory Standards Institute (CLSI) breakpoints: Analysis of agreement. Cureus.

[19] Magiorakos A.-P., Srinivasan A., Carey R.B., Carmeli Y., Falagas M., Giske C., Harbarth S., Hindler J., Kahlmeter G., Olsson-Liljequist B. (2012). Multidrug-resistant, extensively drug-resistant and pandrug-resistant bacteria: An international expert proposal for interim standard definitions for acquired resistance. Clin. Microbiol. Infect..

[20] Hagiya H., Watanabe N., Maki M., Murase T., Otsuka F. (2014). Clinical utility of string test as a screening method for hypermucoviscosity-phenotype K lebsiella pneumoniae. Acute Med. Surg..

[21] Bittencourt S. (2010). FastQC: A Quality Control Tool for High Throughput Sequence Data. Babraham Bioinformatics. https://www.bioinformatics.babraham.ac.uk/projects/fastqc/.

[22] Chen S., Zhou Y., Chen Y., Gu J. (2018). fastp: An ultra-fast all-in-one FASTQ preprocessor. Bioinformatics.

[23] Ewels P., Magnusson M., Lundin S., Käller M. (2016). MultiQC: Summarize analysis results for multiple tools and samples in a single report. Bioinformatics.

[24] Wood D.E., Salzberg S.L. (2014). Kraken: Ultrafast metagenomic sequence classification using exact alignments. Genome Biol..

[25] Choi M., Hegerle N., Nkeze J., Sen S., Jamindar S., Nasrin S., Sen S., Permala-Booth J., Sinclair J., Tapia M.D. (2020). The diversity of lipopolysaccharide (O) and capsular polysaccharide (K) antigens of invasive *Klebsiella pneumoniae* in a multi-country collection. Front. Microbiol..

[26] Seemann T. (2017). Shovill: Faster SPAdes Assembly of Illumina Reads. https://github.com/tseemann/shovill.

[27] Liu P., Li P., Jiang X., Bi D., Xie Y., Tai C., Deng Z., Rajakumar K., Ou H.-Y. (2012). Complete genome sequence of *Klebsiella pneumoniae* subsp. pneumoniae HS11286, a multidrug-resistant strain isolated from human sputum. J. Bacteriol..

[28] Overbeek R., Olson R., Pusch G.D., Olsen G.J., Davis J.J., Disz T., Edwards R.A., Gerdes S., Parrello B., Shukla M. (2014). The SEED and the Rapid Annotation of microbial genomes using Subsystems Technology (RAST). Nucleic Acids Res..

[29] Seemann T. (2016). ABRicate: Mass Screening of Contigs for Antibiotic Resistance Genes.

[30] Alcock B.P., Huynh W., Chalil R., Smith K.W., Raphenya A.R., Wlodarski M.A., Edalatmand A., Petkau A., Syed S.A., Tsang K.K. (2023). CARD 2023: Expanded curation, support for machine learning, and resistome prediction at the Comprehensive Antibiotic Resistance Database. Nucleic Acids Res..

[31] Lam M.M., Wick R.R., Watts S.C., Cerdeira L.T., Wyres K.L., Holt K.E. (2021). A genomic surveillance framework and genotyping tool for *Klebsiella pneumoniae* and its related species complex. Nat. Commun..

[32] Feldgarden M., Brover V., Gonzalez-Escalona N., Frye J.G., Haendiges J., Haft D.H., Hoffmann M., Pettengill J.B., Prasad A.B., Tillman G.E. (2021). AMRFinderPlus and the Reference Gene Catalog facilitate examination of the genomic links among antimicrobial resistance, stress response, and virulence. Sci. Rep..

[33] Liu H., Liu W., Zhou X., Wang X., Huang G. (2025). Genomic characterization of *Klebsiella pneumoniae* clinical isolates from cancer patients: Resistance profiles, virulence factors, and sequence typing. Front. Microbiol..

[34] Carattoli A., Hasman H. (2019). PlasmidFinder and in silico pMLST: Identification and typing of plasmid replicons in whole-genome sequencing (WGS). Horizontal Gene Transfer: Methods and Protocols.

[35] Davis J.J., Gerdes S., Olsen G.J., Olson R., Pusch G.D., Shukla M., Vonstein V., Wattam A.R., Yoo H. (2016). PATtyFams: Protein families for the microbial genomes in the PATRIC database. Front. Microbiol..

[36] Stamatakis A. (2014). RAxML version 8: A tool for phylogenetic analysis and post-analysis of large phylogenies. Bioinformatics.

[37] Chinnasami S., Manickam R. (2023). Classification of the Architecture Using IBM SPSS Statistics.

[38] Idrees E.K., Aldriwesh M.G., Alkhulaifi M.M., Alghoribi M.F. (2025). Systematic review of multidrug-resistant *Klebsiella pneumoniae* in the Arabian Peninsula: Molecular epidemiology and resistance patterns. Front. Microbiol..

[39] Khan E.R., Aung M.S., Paul S.K., Ahmed S., Haque N., Ahamed F., Sarkar S.R., Roy S., Rahman M.M., Mahmud M.C. (2018). Prevalence and molecular epidemiology of clinical isolates of Escherichia coli and *Klebsiella pneumoniae* harboring extended-spectrum beta-lactamase and carbapenemase genes in Bangladesh. Microb. Drug Resist..

[40] Azra, Khan T.A., Ul Haq I., Hinthong W., Campino S., Gohar A., Khan N., Kashif M., Ullah I., Clark T.G. (2025). Antibiotic susceptibility patterns and virulence profiles of classical and hypervirulent *Klebsiella pneumoniae* strains isolated from clinical samples in khyber pakhtunkhwa, pakistan. Pathogens.

[41] Mabrouk S.S., Abdellatif G.R., Abu Zaid A.S., Aboshanab K.M. (2023). New Insights on the Carbapenem-resistant Gram Negative-associated-Infections: Challenges and Opportunities. Arch. Pharm. Sci. Ain Shams Univ..

[42] Peirano G., Chen L., Kreiswirth B.N., Pitout J.D. (2020). Emerging antimicrobial-resistant high-risk *Klebsiella pneumoniae* clones ST307 and ST147. Antimicrob. Agents Chemother..

[43] Matsumura Y., Yamamoto M., Gomi R., Tsuchido Y., Shinohara K., Noguchi T., Nagao M. (2025). Integrating whole-genome sequencing into antimicrobial resistance surveillance: Methodologies, challenges, and perspectives. Clin. Microbiol. Rev..

[44] Azra, Ullah I., Hinthong W., Phelan J.E., Campino S., Haq I.U., Din Z.U., Ahmad S., Rani G.F., Naz A. (2025). Comparative genomic insights into multidrug resistance in classical and hypervirulent K. pneumoniae clinical isolates. Sci. Rep..

[45] Li Y., Kumar S., Zhang L., Wu H., Wu H. (2023). Characteristics of antibiotic resistance mechanisms and genes of *Klebsiella pneumoniae*. Open Med..

[46] Akinola O.T., Dahunsi S.O., Okoh A. (2024). Detection of OMPKs 36/37 porins and other resistance determinants in extended spectrum beta-lactamases (ESBLs)-producing K. pneumoniae. J. Phytomed. Ther..

[47] Yap P.S.-X., Cheng W.-H., Chang S.-K., Lim S.-H.E., Lai K.-S. (2022). MgrB mutations and altered cell permeability in colistin resistance in *Klebsiella pneumoniae*. Cells.

[48] Novelli M., Bolla J.-M. (2024). RND efflux pump induction: A crucial network unveiling adaptive antibiotic resistance mechanisms of gram-negative bacteria. Antibiotics.

[49] Lan P., Lu Y., Fu Y., Yu Y., Zhou J. (2025). Siderophores and beyond: A comprehensive review of iron acquisition in *Klebsiella pneumoniae*. Virulence.

[50] Chaaban T., Mohsen Y., Ezzeddine Z., Ghssein G. (2023). Overview of Yersinia pestis metallophores: Yersiniabactin and yersinopine. Biology.

[51] Singh R.P., Kapoor A., Sinha A., Ma Y., Shankar M. (2025). Virulence factors of *Klebsiella pneumoniae*: Insights into canonical and emerging mechanisms driving pathogenicity and drug resistance. Microbe.

[52] Ali M.R., Yang Y., Dai Y., Lu H., He Z., Li Y., Sun B. (2023). Prevalence of multidrug-resistant hypervirulent *Klebsiella pneumoniae* without defined hypervirulent biomarkers in Anhui, China: A new dimension of hypervirulence. Front. Microbiol..

[53] Dey T., Chakrabortty A., Kapoor A., Warrier A., Nag V.L., Sivashanmugam K., Shankar M. (2022). Unusual hypermucoviscous clinical isolate of *Klebsiella pneumoniae* with no known determinants of hypermucoviscosity. Microbiol. Spectr..

[54] Edward E.A., Mohamed N.M., Zakaria A.S. (2022). Whole genome characterization of the high-risk clone ST383 *Klebsiella pneumoniae* with a simultaneous carriage of bla CTX-M-14 on IncL/M plasmid and bla CTX-M-15 on convergent IncHI1B/IncFIB plasmid from Egypt. Microorganisms.

[55] Wang X., Zhao J., Ji F., Chang H., Qin J., Zhang C., Hu G., Zhu J., Yang J., Jia Z. (2021). Multiple-replicon resistance plasmids of Klebsiella mediate extensive dissemination of antimicrobial genes. Front. Microbiol..

[56] Clegg S., Murphy C.N. (2017). Epidemiology and virulence of *Klebsiella pneumoniae*. Urinary Tract Infections: Molecular Pathogenesis and Clinical Management.

[57] Lin W.-H., Wang M.-C., Tseng C.-C., Ko W.-C., Wu A.-B., Zheng P.-X., Wu J.-J. (2010). Clinical and microbiological characteristics of *Klebsiella pneumoniae* isolates causing community-acquired urinary tract infections. Infection.

[58] Islam M.M., Satici M.O., Eroglu S.E. (2024). Unraveling the clinical significance and prognostic value of the neutrophil-to-lymphocyte ratio, platelet-to-lymphocyte ratio, systemic immune-inflammation index, systemic inflammation response index, and delta neutrophil index: An extensive literature review. Turk. J. Emerg. Med..

