# Supplementary material for: Clinical and Genomic Characterization of High-Risk Multidrug-Resistant Klebsiella pneumoniae Lineages in Pakistan
Source: Microorganisms. 2026 Jul 2;14(7):1462. doi: 10.3390/microorganisms14071462 (PMC13413983; doi:10.3390/microorganisms14071462)
Supplement: Supplementary file 1 [file microorganisms-14-01462-s001.zip › microorganisms-4331566-supplementary.pdf]

microorganisms

MDPI

*Article*

# Clinical and Genomic Characterization of High-Risk Multidrug-Resistant *Klebsiella pneumoniae* Lineages in Pakistan

## Supplementary Data

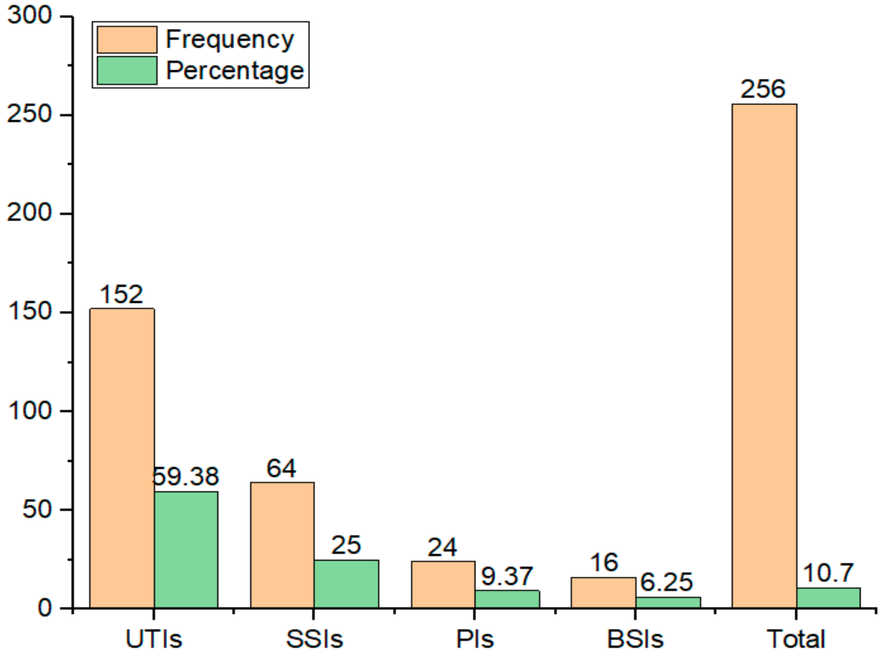


**Figure S1:** Various infections and sample types retrieved from *K. pneumoniae* isolates, Urinary Tract Infections=UTIs, Bloodstream Infections= BSIs, Pulmonary Infections= PIs, and Surgical Site Infections= SSIs

**Table S1:** Correlation of Demographic and Comorbid Factors with *K. pneumoniae* Infection

| Variable | Groups | Study Population | Negative (%) | Positive (%) | *p*-Value |
|---|---|---|---|---|---|
| Gender | Male | 750 | 658(64.06) | 92(35.94) | 0.001 |
| | Female | 1650 | 1486(35.94) | 164(64.06) | |
| Age wise Groups | <1 year | 112 | 100 (4.17%) | 12 (4.68) | 0.002 |
| | 1–10 years | 263 | 235 (9.79) | 28 (10.93) | |
| | 11–20 years | 150 | 134 (5.58) | 16 (6.25) | |
| | 21–30 years | 600 | 536 (22.33) | 64 (25) | |
| | 31–40 years | 225 | 201 (8.38) | 24 (9.3) | |
| | 41–50 years | 300 | 268 (11.17) | 32 (12.5) | |
| | 51–60 years | 352 | 316 (13.17) | 36 (14.0) | |
| | 61–70 years | 324 | 288 (12.0) | 36 (14.0) | |
| | 71+ years | 74 | 66 (2.75) | 8 (3.1) | |
| | Peshawar | 1604 | 1428 (59.5) | 172 (67.18) | |
| | Abbottabad | 370 | 333 (13.88) | 40 (15.62) | |
| | Khyber | 112 | 95 (3.96) | 12 (4.68) | |
| | Charsadda | 120 | 112 (4.67) | 12 (4.68) | |

| | | | | | |
|---|---|---|---|---|---|
| Regional distribution | Mohmand | 75 | 67 (2.79) | 8 (3.12) | 0.003 |
| | Mardan | 38 | 34 (1.42) | 4 (1.56) | |
| | Kurram | 40 | 36 (1.50) | 4 (1.56) | |
| | Nowshera | 43 | 39 (1.63) | 4 (1.56) | |
| Laboratory Findings | Leucocytosis | 2144 | 85 (33.2) | 171 (66.8) | 0.005 |
| | Neutrophilia | 2144 | 85 (33.2) | 192 (75.0) | 0.002 |
| | Raised CRP | 2144 | 0 (0.0) | 256 (100) | 0.001 |
| | Raised ALT | 2144 | 228 (89.1) | 28 (10.9) | 0.061 |
| | Raised Urea | 2144 | 171 (66.8) | 85 (33.2) | 0.034 |
| | Raised Creatinine | 2144 | 114 (44.6) | 142 (55.4) | 0.048 |
| Comorbidities | Diabetes Mellitus | 2144 | 140 (54.7) | 116 (45.3) | 0.042 |
| | Hypertension | 2144 | 172 (67.2) | 84 (32.8) | 0.078 |
| | Chronic Kidney Disease | 2144 | 188 (73.4) | 68 (26.6) | 0.015 |
| | Coronary Heart Disease | 2144 | 200 (78.1) | 56 (21.9) | 0.006 |
| | Interstitial Lung Disease | 2144 | 232 (90.6) | 24 (9.4) | 0.001 |

**Table S2:** Factors associated with *K. pneumoniae* infection in the current study (n = 2400)

| **Characteristics** | **Univariate Analysis** | | | **Multivariate Analysis** | | |
|---|---|---|---|---|---|---|
| | **OR** | **95% CI** | **p-Value** | **aOR** | **95% CI** | **p-Value** |
| **Gender** | | | | | | |
| Female vs. Male | 1.72 | 1.32–2.24 | 0.001 | 1.65 | 1.25–2.19 | <0.001 |
| **Age Group (ref: <1 year)** | | | | | | |
| 21–30 years | 1.37 | 1.01–1.86 | 0.041 | 1.41 | 1.02–1.95 | 0.036 |
| 51–60 years | 1.44 | 1.06–1.96 | 0.021 | 1.48 | 1.08–2.03 | 0.014 |
| 61–70 years | 1.44 | 1.05–1.99 | 0.023 | 1.45 | 1.04–2.02 | 0.027 |
| **Region (ref: other districts)** | | | | | | |
| Peshawar | 1.47 | 1.12–1.93 | 0.005 | 1.43 | 1.10–1.86 | 0.006 |
| Abbottabad | 1.33 | 0.94–1.89 | 0.102 | 1.29 | 0.91–1.83 | 0.139 |
| **Laboratory Findings** | | | | | | |
| Leucocytosis | 2.21 | 1.55–3.15 | 0.005 | 2.25 | 1.55–3.25 | <0.001 |
| Neutrophilia | 3.88 | 2.67–5.64 | 0.002 | 3.95 | 2.71–5.76 | <0.001 |
| Raised CRP level | 15.80 | 7.99–31.24 | 0.001 | 14.50 | 7.20–29.20 | <0.001 |
| Raised Urea | 1.55 | 1.11–2.17 | 0.034 | 1.55 | 1.12–2.17 | 0.008 |
| Raised Creatinine | 1.43 | 1.01–2.03 | 0.048 | 1.48 | 1.06–2.07 | 0.019 |
| Raised ALT | 0.72 | 0.50–1.03 | 0.061 | 0.75 | 0.52–1.08 | 0.114 |
| **Comorbidities** | | | | | | |
| Diabetes Mellitus | 1.52 | 1.10–2.10 | 0.042 | 1.52 | 1.10–2.10 | 0.012 |
| Hypertension | 0.81 | 0.61–1.09 | 0.078 | 0.86 | 0.63–1.16 | 0.308 |
| Chronic Kidney Disease | 0.65 | 0.45–0.94 | 0.015 | 0.68 | 0.48–0.97 | 0.032 |
| Coronary Heart | 0.62 | 0.44–0.89 | 0.006 | 0.71 | 0.49–1.02 | 0.066 |

| | | | | | | |
|---|---|---|---|---|---|---|
| Disease | | | | | | |
| Interstitial Lung Disease | 0.34 | 0.20–0.57 | 0.001 | 0.38 | 0.24–0.61 | <0.001 |

**Table S3.** Clinical, phenotypic, and genomic characteristics of 18 *K. pneumoniae* isolates selected for WGS

| Isolate ID | Specimen Source | Clinical Diagnosis | String Test | Meropenem Ressitance | Virulence Category | ST | MDR /XDR | Viruilence Score* | Resistrance Score* |
|---|---|---|---|---|---|---|---|---|---|
| Kp1210 | Pus | Abscess | Negative | S | Non-hypermucoid | 151 | MDR | 1 | 0 |
| Kp1215 | Pus | Abscess | Negative | R | Non-hypermucoid | 1391-1LV | MDR | 0 | 1 |
| Kp1221 | Urine | UTI | Negative | S | Non-hypermucoid | 376 | MDR | 1 | 1 |
| Kp1203 | Urine | UTI | Negative | R | Non-hypermucoid | 147 | XDR | 1 | 2 |
| Kp1211 | Pus | Wound | Negative | R | Non-hypermucoid | 870 | MDR | 1 | 3 |
| Kp1224 | Pus | Wound | Negative | R | Non-hypermucoid | 45 | XDR | 1 | 1 |
| Kp1222 | Urine | Pus | Wound | S | Non-hypermucoid | 147 | XDR | 1 | 1 |
| Kp1200 | Pus | Abscess | Positive | S | Hypermucoid | 147 | XDR | 1 | 1 |
| Kp1199 | Pus | Wound | Positive | S | Hypermucoid | 147 | XDR | 1 | 1 |
| Kp1186 | Pus | Cellulitis | Positive | S | Hypermucoid | 2450-1LV | XDR | 0 | 0 |
| Kp1187 | Pus | Cellulitis | Positive | R | Hypermucoid | 37 | MDR | 0 | 1 |
| Kp1190 | Pus | Wound | Positive | R | Hypermucoid | 37 | XDR | 0 | 1 |
| Kp1191 | Pus | Abscess | Positive | S | Hypermucoid | 1310 | MDR | 1 | 0 |
| Kp1192 | Pus | Abscess | Positive | S | Hypermucoid | 2629 | MDR | 0 | 0 |
| Kp1193 | Pus | Cellulitis | Positive | S | Hypermucoid | 2629 | MDR | 0 | 0 |
| Kp1196 | Pus | Abscess | Positive | S | Hypermucoid | 2629 | MDR | 0 | 0 |
| Kp1194 | Blood | Septicemia | Positive | R | Hypermucoid | 147 | XDR | 1 | 2 |
| Kp1219 | Pus | Wound | Negative | S | Non-hypermucoid | 147 | MDR | 1 | 0 |

***Kleborate Virulence Score:** 0 indicate no virulence loci detected; 1 indicates yersiniabactin only.
***Kleborate Resistance Score:** 0 show no ESBL or carbapenemase detected; 1 means ESBL detected; 2 indicates carbapenemase detected; 3 refers to carbapenemase plus colistin-resistance determinant detected.

**Table S4:** *De novo genome assembly of K. pneumoniae isolates (n=18)*

| Genome ID | Contigs count | Genome Length | GC Content (%) | L50 | N50 | CDS |
|---|---|---|---|---|---|---|
| Kp1210 | 128 | 5717468 | 56.9683 | 5 | 287843 | 5658 |
| Kp1215 | 170 | 5553382 | 57.2392 | 4 | 448857 | 5465 |
| Kp1221 | 143 | 5498315 | 57.1758 | 8 | 197431 | 5448 |
| Kp1203 | 224 | 5954829 | 56.6781 | 10 | 235629 | 6058 |
| Kp1211 | 256 | 6876792 | 57.4966 | 16 | 245379 | 5654 |
| Kp1224 | 630 | 6443909 | 58.2317 | 14 | 127939 | 6795 |
| Kp1222 | 233 | 5953677 | 56.6768 | 9 | 232237 | 6057 |
| Kp1200 | 251 | 5957613 | 56.6777 | 10 | 214633 | 6061 |
| Kp1199 | 264 | 5959172 | 56.6749 | 10 | 183179 | 6090 |
| Kp1186 | 136 | 5260495 | 57.5196 | 5 | 451750 | 5139 |
| Kp1187 | 154 | 5368084 | 57.4147 | 6 | 350969 | 5266 |
| Kp1190 | 158 | 5369118 | 57.4093 | 6 | 350969 | 5264 |
| Kp1191 | 251 | 5650939 | 57.1101 | 9 | 238045 | 5672 |
| Kp1192 | 82 | 5405504 | 57.2854 | 5 | 442842 | 5295 |
| Kp1193 | 99 | 5423902 | 57.258 | 5 | 376578 | 5334 |
| Kp1196 | 89 | 5405795 | 57.2848 | 4 | 373249 | 5307 |
| Kp1194 | 103 | 5700694 | 56.8848 | 8 | 267654 | 5651 |
| Kp1219 | 236 | 5443779 | 56.7617 | 11 | 258796 | 5783 |

**Table S5:** Mutational analysis of AMR genes resistant isolates of *K. pneumoniae*

| Gene | Mutation | Isolates Carrying Mutation | Frequency (n=18) | Associated Antimicrobial Class |
|---|---|---|---|---|
| *gyrA* | S83I | All | 18 | Fluoroquinolones |
| | F862I | All | 18 | Fluoroquinolones |
| *parC* | S80I | All | 18 | Fluoroquinolones |
| | N304S | Kp1194, Kp1224, Kp1219, Kp1200,Kp1199, Kp1222, Kp1203 | 7 | Fluoroquinolones |
| | S681A | All | 18 | Fluoroquinolones |
| | V678E | All | 18 | Fluoroquinolones |
| *mgrB* | M1V | All | 18 | Colistin |
| *pmrB* | T246A | Kp1194, Kp1203, Kp1222, Kp1224, Kp1190, Kp1200, Kp1221, Kp1187, Kp1186Kp1210, Kp1199, Kp1191 | 12 | Colistin |
| | T240M | Kp1191 | 1 | Colistin |
| *arnC* | S30T | All | 18 | Colistin |
| | K322Q | Kp1186, Kp1191 | 2 | Colistin |
| *lapB* | N212T | All | 18 | Colistin |
| *lpxM* | S253G | Kp1194, Kp1196, Kp1193, Kp1192, Kp1191, Kp1224, Kp1200, Kp1210, Kp1215, Kp1221, | 14 | Colistin |

| | | | | |
|---|---|---|---|---|
| | | Kp1199, Kp1190, Kp1219, Kp1211 | | |
| *ramR* | M1V | All | 18 | Tigecycline |
| *ftsI* | V375A | All | 18 | β-lactams |
| *cirA* | A210T | Kp1191 | 1 | Cefiderocol |
| | D558N | Kp1191 | 1 | Cefiderocol |
| *ompK36* | N221H | Kp1186, Kp1193, Kp1192, Kp1211, Kp1203, Kp1215, Kp1222, Kp1199, Kp1190, Kp1210, Kp1191, Kp1196, Kp1200, Kp1219 | 14 | Carbapenems |
| | H349R | Kp1186, Kp1196, Kp1193, Kp1192, Kp1211, Kp1210, Kp1215,Kp1194 | 8 | Carbapenems |
| | V178P | Kp1194, Kp1211 | 2 | Carbapenems |
| | V269P | Kp1194, Kp1211, Kp1221, Kp1203, Kp1186, Kp1187 | 6 | Carbapenems |
| | A183T | Kp1194, Kp1211, Kp1221, Kp1204, Kp1187, Kp1215 | 6 | Carbapenems |
| | A233T | Kp1194, Kp1211, Kp1221, Kp1204, Kp1187, Kp1216 | 6 | Carbapenems |
| | D344E | Kp1194, Kp1211, Kp1221, Kp1206, Kp1224, Kp1190 | 6 | Carbapenems |
| | D349E | Kp1194, Kp1211, Kp1221, Kp1190, Kp1207, Kp1224, | 6 | Carbapenems |
| | I315L | Kp1194, Kp1211, Kp1221, Kp1208, Kp1187, Kp1224 | 6 | Carbapenems |
| | L225N | Kp1194, Kp1211, Kp1221, Kp1204, Kp1187, Kp1216 | 6 | Carbapenems |
| | L307I | Kp1186, Kp1193, Kp1192, Kp1211, Kp1203, Kp1215, Kp1222, Kp1199, Kp1190, Kp1210, Kp1191, Kp1196, Kp1200, Kp1219 | 14 | Carbapenems |
| | S346D | Kp1194, Kp1211, Kp1221, Kp1211, Kp1224, Kp1190 | 6 | Carbapenems |
| | S230R | Kp1194, Kp1211, Kp1221, Kp1212, Kp1187, Kp1190 | 6 | Carbapenems |
| | T192G | Kp1194, Kp1211, Kp1221, Kp1213, Kp1190, Kp1224 | 6 | Carbapenems |
| | Y201F | Kp1186, Kp1193, Kp1192, Kp1211, Kp1203, Kp1222, Kp1199, Kp1190, Kp1210, Kp1191, Kp1196, Kp1200, Kp1219 | 13 | Carbapenems |
